# Predominance and high diversity of genes associated to denitrification in metagenomes of subantarctic coastal sediments exposed to urban pollution

**DOI:** 10.1371/journal.pone.0207606

**Published:** 2018-11-29

**Authors:** Priscila A. Calderoli, Fernando J. Espínola, Hebe M. Dionisi, Mónica N. Gil, Janet K. Jansson, Mariana Lozada

**Affiliations:** 1 Laboratorio de Microbiología Ambiental, Centro para el Estudio de Sistemas Marinos, CONICET, Puerto Madryn, Chubut Province, Argentina; 2 Laboratorio de Oceanografía Química y Contaminación de Aguas, Centro para el Estudio de Sistemas Marinos, CONICET, Puerto Madryn, Chubut Province, Argentina; 3 Laboratorio de Química General y Análisis de Elementos, CCT CONICET CENPAT, Puerto Madryn, Chubut Province, Argentina; 4 Biological Sciences Division, Earth and Biological Sciences Directorate, Pacific Northwest National Laboratory, Richland, Washington, United States of America; The University of Akron, UNITED STATES

## Abstract

The aim of this work was to characterize the microbial nitrogen cycling potential in sediments from Ushuaia Bay, a subantarctic environment that has suffered a recent explosive demographic growth. Subtidal sediment samples were retrieved in triplicate from two urban points in the Bay, and analyzed through metagenomic shotgun sequencing. Sequences assigned to genes related to nitrification, nitrate reduction and denitrification were predominant in this environment with respect to metagenomes from other environments, including other marine sediments. The *nos*Z gene, responsible for nitrous oxide transformation into di-nitrogen, presented a high diversity. The majority of NosZ sequences were classified as Clade II (atypical) variants affiliated to different bacterial lineages such as Bacteroidetes, Chloroflexi, Firmicutes, Proteobacteria, Verrucomicrobia, as well as to Archaea. The analysis of a fosmid metagenomic library from the same site showed that the genomic context of atypical variants was variable, and was accompanied by distinct regulatory elements, suggesting the evolution of differential ecophysiological roles. This work increases our understanding of the microbial ecology of nitrogen transformations in cold coastal environments and provides evidence of an enhanced denitrification potential in impacted sediment microbial communities. In addition, it highlights the role of yet overlooked populations in the mitigation of environmentally harmful forms of nitrogen.

## Introduction

Sediment microbial communities are key components of coastal ecosystems, driving fundamental biological transformations such as organic matter decomposition and nutrient cycling, and therefore contributing to ecosystem sustainability [1–3]. A number of interconversions of nitrogen (N) species within the N-cycle occur across oxygen-fluctuating conditions near the sediment surface [3]. There are two major sources of nitrate (NO_3_^-^) and nitrite (NO_2_^-^) for marine sediments: the supply from the overlying water by means of diffusion, and nitrification (oxidation of ammonium, NH_4_^+^) occurring in the oxic layer of the sediment [4,5]. Coastal sediments significantly contribute to denitrification, being responsible for a large fraction of the marine di-nitrogen (N_2_) release into the atmosphere [6,7]. Besides N_2_ release, nitrification coupled to (incomplete) denitrification results in the emission of nitrous oxide (N_2_O), a greenhouse gas with 300 times the global warming potential of carbon dioxide (CO_2_), which is estimated to contribute 10% to climate change each year [8,9] and to be the dominant ozone-depleting substance [10]. Another source of N_2_O is as a by-product of the dissimilatory NO_3_^-^ reduction to NH_4_^+^ (DNRA), a competing nitrate-reduction process that can sometimes be dominant, governing the fate of NO_3_^-^ in coastal ecosystems [11,12].

The capability for denitrification is widely spread among bacteria. Many facultative anaerobes use NO_3_^-^ due to its high reduction potential, which makes it a good electron acceptor under anaerobic conditions [13,14]. However, as the denitrification pathway includes multiple sequential reactions across a wide range of redox states, few organisms are capable of carrying out the complete process, whereas others can only reduce the N_2_O to N_2_ [14]. Complete denitrification requires the combined action of multiple members, denoting the role of community structure in controlling N_2_O emission levels [15–17]. The enzymes that catalyze these reactions are encoded by the genes *nap*A and *nar*G, *nir*K and *nir*S, *nor*B and *nos*Z, for NO_3_, nitrite (NO_2_), nitric oxide (NO) and N_2_O reduction, respectively [13]. These genes have been widely used as predictors of the capability (“biomarkers”) of the different steps of denitrification [18–20]. The genes *nap*A and *nar*G are also used as biomarkers for DNRA, together with *nir*B and *nrf*A [21–24]. Although useful for uncovering new gene variants and their roles in different ecosystems, amplicon-based environmental surveys of biomarker genes still cannot cover the whole array of microorganisms carrying N-cycle genes, such as *nir*K-type denitrifiers [25]. Shotgun metagenomics surveys can provide not only valuable information on the phylogenetic diversity, but can also allow the comparison of the relative abundance of the biomarker genes for the different N-cycling subprocesses, providing detailed information of the N-cycling patterns in microbial communities [17,19,26–28].

**Fig 1:**
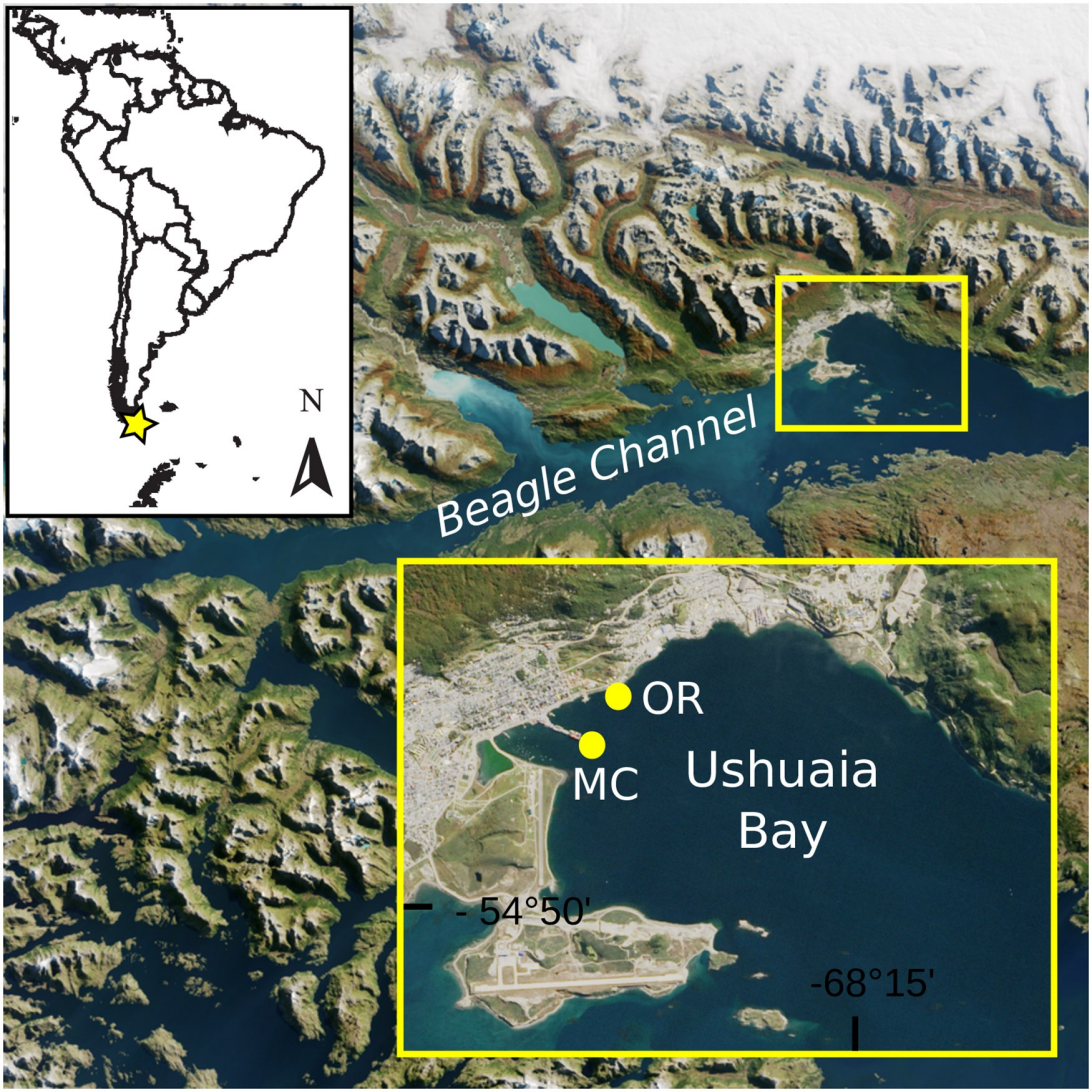
Sampling sites. **A-** Sampling location in Ushuaia Bay (Tierra del Fuego Island, Argentina). **B-** Location of the two sampling sites within Ushuaia Bay. For further details, see S1 Table. Satellite image was obtained from NASA Earth Observatory (https://earthobservatory.nasa.gov).

Ushuaia Bay (Tierra del Fuego Island, Argentina) is a semi-enclosed coastal system characterized by low-energy shores, situated within the Beagle Channel, in the southernmost tip of South America. It has recently been subjected to multiple impacts due to the fast population growth of Ushuaia city, including the discharge of untreated urban and industrial effluents as well as hydrocarbon pollution from various sources [29–32]. In this work, two sites within Ushuaia Bay have been analyzed, the Commercial Pier (MC) and Orion Plant jetty (OR), both of them placed on the coast of Ushuaia city. Despite being located in the same environment separated by only 500 m (Fig 1), the two sampled sites are locally exposed to different anthropogenic activities and have been classified into different water quality zones [30]. MC site receives intense maritime traffic, and is located in the Northwestern zone of the Bay, an area impacted by urban effluents either directly from near discharges or indirectly from its connection with Encerrada Bay. Encerrada Bay is a highly eutrophicated, semi-enclosed system that constantly receives labile, carbon-rich compounds, as well as nutrients from untreated urban effluents [29,30]. On the other hand, at OR site, organic matter content is mainly attributed to chronic hydrocarbon pollution due to the activities carried out at the oil jetty [30,33]. In this environmental context, the capability for a complete denitrification process in Ushuaia Bay sediments could represent a potential N loss from the ecosystem, alleviating the effects of the excessive nutrient load in this environment and turning it into in a non-toxic form. Evidence of nitrate consumption has been reported in Ushuaia Bay sediments [30], although no information was available up to now on the microbial populations involved in N-cycling in this system, or in other high-latitude coastal sediments impacted by anthropogenic pressure.

In this work, we analyzed the microbial N-cycling potential in Ushuaia Bay sediments, in the search for genetic patterns for different subprocesses within this cycle in this chronically impacted ecosystem. Through the analysis of a metagenomic dataset, we found nitrification and denitrification biomarker genes to be dominant with respect to the ones from other subprocesses, as well as overrepresented with respect to other metagenomes from diverse environments. A high diversity of sequences assigned to *nos*Z (coding for nitrous-oxide reductase, responsible for the depuration of N2O) was observed, mainly composed of atypical variants.

## Materials and methods

### Sediment sampling and chemical analysis

Subtidal sediments (11.3–12.3 m bathymetry) were collected from the coast of Ushuaia Bay (Tierra del Fuego Island, Argentina), in December of 2008. Samples (0–5 cm) were obtained by scuba-diving using cores and stored at -80 C and -20 C for DNA analysis and chemical analysis, respectively. Each of the studied sites was sampled in triplicate: (a) the city Commercial pier (MC, 54° 48.656′ S 68° 17.731′ W, samples ARG01-ARG03) and (b) Orion Plant oil jetty (OR, 54° 48.256′ S 68° 17.296′ W, samples ARG04-ARG06) (Fig 1 and S1 Table). No specific permissions were required for these locations, as they are public urban sites and none of them belong to protected areas or involved endangered species. Metadata (depth, temperature, pH and salinity in the sediment-water interface) were measured *in-situ* with a multiparameter instrument. Total organic matter content (TOM) was obtained by calculating weight loss after calcination (450 °C, 4 h), and total ammonium (NH_4_^+^) content was obtained by following the method described by Strickland and Parsons (1972) [34] after extraction with 2N potassium chloride (KCl).

### Metagenomic DNA extraction and sequencing

Total community DNA was extracted from each core sample using a modified beadbeating protocol in a FastPrep Instrument (MP Biomedicals), as previously described [35]. Briefly, approximately 0.5 g of sediment was added to 2.0 ml tubes containing beads and extraction buffer (10% CTAB in 1 M NaCl, 0.1 M (NH_4_)_2_SO_4_ and 0.5 ml phenol:chloroform:isoamylalcohol 25:24:1), and shaken in a FastPrep Instrument (MP Biomedicals), followed by chloroform extraction and precipitation in a PEG 6000/1.6 M NaCl solution. Extractions were purified using an AllPrep DNA/RNA kit (Qiagen), and quantified using Quant-iT dsDNA HS assay kit (Invitrogen) according to the manufacturer’s instructions. Metagenomic shotgun sequencing was performed with Illumina HiSeq 2000 platform (2 × 150-bp paired end reads, one lane per sample). Sequencing was carried out at the facilities of the United States Department of Energy (DOE) Joint Genome Institute (JGI, http://genome.jgi.doe.gov) and annotated using the Integrated Microbial Genomes & Microbiomes (IMG/M) pipeline [36]. Further information about sampling, sequencing and processing of the samples can be found elsewhere [33,37,38].

### Bioinformatic analysis of metagenomic sequences

Functional annotation performed with the IMG/M pipeline was used for metagenomic sequence analysis, as described below. KEGG orthology (KO) identifiers [39] were used to retrieve coding sequences (CDS) potentially associated to key subprocesses within N-cycle: N-fixation, nitrification, assimilatory nitrite reduction, dissimilatory nitrate-nitrite reduction, and denitrification. The following KO identifiers were used: K02588 (NifH, nitrogenase iron protein), K02586 (NifD, nitrogenase molybdenum-iron protein alpha chain), K02591 (NifK, nitrogenase molybdenum-iron protein beta chain), K10944 (AmoA, ammonia monooxygenase subunit A), K10945 (AmoB, ammonia monooxygenase subunit B), K10535 (Hao, hydroxylamine dehydrogenase), K00362 (NirB, nitrite reductase NAD(P)H large subunit), K00366 (NirA, ferredoxin-nitrite reductase), K03385 (NrfA, nitrite reductase cytochrome c-552), K02567 (NapA, periplasmic nitrate reductase), K00370 (NarG, membrane-bound nitrate reductase alpha subunit, and NxrA, nitrite oxidoreductase alpha subunit), K15864 (NirS, cytochrome cd1-type nitrite reductase), K00368 (NirK, Cu-containing nitrite reductase), K04561 (NorB, nitric oxide reductase subunit B) and K00376 (NosZ, nitrous oxide reductase). The abundance of each of the corresponding biomarker genes was estimated by calculating the proportion of amino acid sequences assigned to the KO of interest (estimated gene copies, assembled and unassembled metagenomes, as retrieved from IMG/M) and normalized with respect to the total number of sequences assigned to KOs in each metagenome. The corresponding protocol can be found in protocols.io: dx.doi.org/10.17504/protocols.io.u32eyqe. For K00370, sequences corresponding to NarG or NxrA were further discriminated by phylogenetic placement and totals for each gene were calculated using in-house R scripts (available upon request). Since sequences affiliated with NxrA represented less than 2% of the total sequences assigned to K00370, they were not taken into account in further analyses. For sequences assigned to genes related with the anaerobic ammonium oxidation (anammox) process, for which no KO identifier is yet available, a Hidden Markov Model (HMM) [40] was built for the *alpha* subunit of the hydrazine synthase (HzsA). The HMM was used for detecting homologous sequences in the metagenomes, searching against all the deduced amino acid sequences from the assembled and unassembled fractions. The HzsA sequences used for building the HMM were (Genbank accession numbers): KKO20072, AEW50029, AEW50031, AEW50032, AEW49994 and AEW50040. Abundances of metagenomic sequences were further corrected by taking into account the average read depth for scaffolds in the assembled fraction, and normalized with respect to the total number of coding sequences in each metagenome. For comparative analysis of the N-cycling potential in Ushuaia Bay sediments with respect to other natural environments, the same procedure was used to retrieve the biomarker genes from 127 selected metagenomes available at IMG/M, representative of different habitats. For details of these samples, see S2 Table. Metagenomic sequences with homology to transcriptional regulators were detected using the blastp algorithm against the assembled and unassembled fraction of the metagenomes, with a threshold E-value of 10^−10^ and the following accession numbers as query: (i) NosR, EAQ26534 (nitrous-oxide reductase transcriptional activator NosR [*Roseovarius* sp. 217]); (ii) NorR, WP_114134079 (nitric oxide reductase transcriptional regulator NorR [*Cupriavidus necator*]); (iii) Rrf2 family, EAR00840 (transcriptional regulator, BadM/Rrf2 family protein [Flavobacteriales bacterium HTCC2170]) and WP_013305448 (Rrf2 family transcriptional regulator [*Maribacter* sp. HTCC2170]); (iv) NnrR-like NNR family, WP_108881854 (Crp/Fnr family transcriptional regulator [*Anderseniella* sp. Alg231-50]); (v) FnrP-like NNR family, EDN73362.1 (ferric nitrate regulator FnrP [*Mannheimia haemolytica* PHL213]; (vi) NarR-like NNR family, AAK61312 (transcriptional activator NarR [*Paracoccus pantotrophus*]); (vii) NnrS, YP_353405.1 (NnrS [*Rhodobacter sphaeroides* 2.4.1]); (viii) NirI, CAA04667 (NirI protein [*Paracoccus denitrificans* PD1222]).

### Ecological and statistical analyses

Differences in the relative abundances of the biomarker genes in the metagenomes from the two sampling sites (MC and OR) were analyzed by Welch’s Two Sample t-test using R (https://cran.r-project.org/). Correlation tests between environmental variables were performed in R environment. Clustering analysis based on relative abundance of biomarker genes across samples was performed using average linkage algorithm in R-package *vegan* (https://cran.r-project.org/web/packages/vegan/index.html). The N-cycling biomarkers contributing to the observed similarity in community patterns were identified using similarity percentage analysis (SIMPER, [41]) based on Bray-Curtis dissimilarities among samples, developed in *vegan*.

The metagenomic sequences coding for putative NirK and NosZ enzymes were clustered *de novo* into OTUs (operational taxonomic units) using CD-HIT Suite program [42]. Identity threshold values of 83 and 80% at the amino acid level were used to defined OTUs for NirK and NosZ, respectively, as this identity was found to correlate with natural clades [43,44]. The datasets were rarefied to 746 and 813 amino acid sequences per sample (NirK and NosZ, respectively) for the calculation of ecological estimators (Chao1 and Shannon- Wiener, H’) at the same sequencing effort. Diversity estimators for each gene were calculated in QIIME [45]. Significant differences in diversity were evaluated by Welch’s Two Sample t-test in R environment.

### Phylogenetic analyses

Metagenomic sequences were analyzed phylogenetically with the Evolutionary Placement Algorithm, in RaxML [46]. Briefly, metagenomic sequences were aligned into a fixed reference alignment with ClustalX [47] and further placed in a reference tree built by Maximum Likelihood method. For NirK sequences, the reference alignment was constructed with the NirK reference database proposed by Helen *et al*., 2016 [25]. In the case of NosZ, full-length NosZ sequences were retrieved from the *fungene* database (http://fungene.cme.msu.edu/), excluding environmental sequences. Both databases were further manually curated, resulting in 267 (NirK) and 444 (NosZ) amino acid sequences representative of the phylogenetic diversity of each gene. Phylogenetic placements were visualized using pplacer/guppy [48].

### Gene context analysis of *nos*Z genes

A metagenomic fosmid library carrying inserts of approximately 40 kb was constructed from intertidal sediments from the same environment (OR sample, [49]). The library was fully sequenced by shotgun sequencing using Illumina HiSeq 1500 (Indear, Argentina). Quality-based preprocessing of the library reads was done using PRINSEQ (stand alone lite version, http://prinseq.sourceforge.net/), with the following options: 3’ trimming based on quality score = 25, dereplicating exact duplicates, and filtering reads with more than 2 ambiguous bases. Reads assigned to the fosmid vector and to *Escherichia coli* (host) were eliminated by mapping against the corresponding sequences (EU140752, cloning vector pCC2FOS, and NC_00913.3, *E*. *coli* str. K-12 substr. MG1655, complete genome) with Bowtie [50] and the *vecscreen* tool in JCVI utility library (https://github.com/tanghaibao/jcvi). Preprocessed reads were assembled using Ray-Meta [51], and genes were predicted using MetaGeneMark [52]. A KO search using K00376 was used to identify putative NosZ in the library, and the complete nucleotide sequences from the corresponding scaffolds were further retrieved. The identified sequences were analyzed phylogenetically as described above, and phylogenetic trees were constructed by Neighbor Joining in ClustalX [47]. Scaffolds were taxonomically assigned using PhyloPythiaS [53], annotated with the RAST pipeline (http://rast.nmpdr.org/), manually curated and visualized in Trebol (http://es.imo-chile.cl/software/trebol.html).

### Accession numbers

The metagenomes analyzed in this work were deposited at IMG with genome IDs 3300000122, 3300000242, 3300000118, 3300000121, 3300000131 and 3300000125 (ARG01-ARG06, respectively). The scaffolds carrying NosZ genes selected from the metagenomic library are available at Genbank (accession numbers MF990890-MF990904).

## Results

### N-cycling genetic signatures in high-latitude coastal sediments

The two sites analyzed within Ushuaia Bay are distanced by 500 m (Fig 1). MC site is located next to the Commercial Pier of Ushuaia city, while OR site is located next to an oil jetty (Orion Plant) [33]. In general, the physicochemical characteristics were comparable between sites (S1 Table). In addition, ammonium (NH_4_^+^) values were comparable to those measured in sediments of this bay in a previous study [30]. In particular, ARG06 (from OR site) showed the higher values of total organic matter (TOM) and NH_4_^+^ concentrations (S1 Table). Organic matter and NH_4_^+^ content were highly correlated with hydrocarbon concentration, previously measured in OR samples [38] (r = 0.989, p-value = 0.002 and r = 0.90, p-value = 0.04, for TOM and NH_4_^+^, respectively).

The metagenomic dataset generated by shotgun sequencing contained 2.39 x 10^7^ to 9.69 x 10^7^ protein-coding sequences. A total of 201,414 sequences were functionally assigned to 15 genes encoding N-cycling enzymes, including NifH, NifD and NifK (nitrogen fixation), AmoA, AmoB and Hao (nitrification), NirA (assimilatory nitrite reduction), NarG and NapA (nitrate reduction, dissimilatory), NrfA and NirB (dissimilatory nitrite reduction to ammonium, DNRA), NirS, NirK, NorB and NosZ (denitrification), and HszA (anammox) (S2 Table). With the exception of NirS, all sequences encoding N-metabolizing enzymes were detected in the metagenomes (S2 Table).

**Fig 2:**
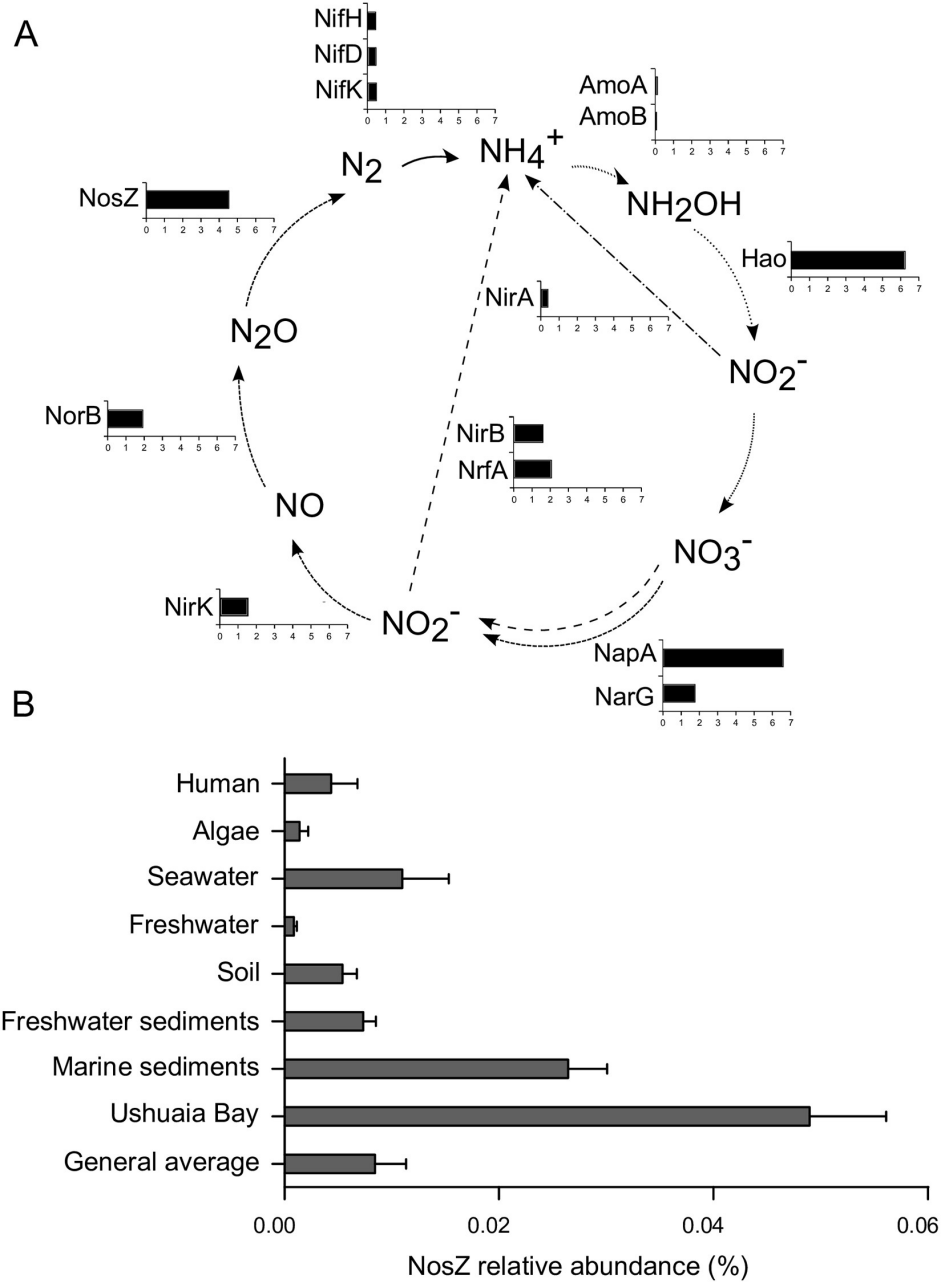
Abundance of putative genes related to the N-cycle in metagenomes from Ushuaia Bay sediments, with respect to metagenomes from different environments. Metagenomic sequences were annotated through the IMG/M pipeline and identified using KEGG orthology (KO) identifiers. Abundances were retrieved as “estimated gene copies” (assembled and unassembled fractions), normalized to the total number of sequences assigned to KOs. For details of the 127 metagenomes used for comparisons, see S2 Table. (A) Relative abundance of N-cycling biomarkers in Ushuaia Bay, with respect to average in metagenomes from different environments. The scale in barplots is fold overrepresentation (abundance in Ushuaia Bay sediments/average abundance across all metagenomes). Arrows:–––nitrogen fixation, …. nitrification, -.-.-. nitrite assimilation,–––DNRA, - - - denitrification. (B) Relative abundance of the sequences assigned to NosZ, discriminated by type of environment.

Sequences assigned to genes related to nitrification, nitrate/nitrite reduction, and denitrification were more abundant than the biomarkers for other subprocesses of the nitrogen cycle (S1 Fig). For instance, sequences coding for NapA, NarG (nitrate reduction) and NosZ (nitrous oxide reduction) were 25 times higher on average than sequences from enzymes participating in nitrogen fixation, such as NifH, NifD and NifK (S2 Table). Accordingly, their corresponding KEGG pathway modules were also predominant among the N metabolism modules in these metagenomes: M00530 (“Dissimilatory nitrate reduction, nitrate to ammonia”) represented 0.44 ± 0.06% of the estimated gene copies, and M00529 (“Denitrification, nitrate to nitrogen”) represented 0.54 ± 0.10%, while M00528 (“Nitrification”) was only 0,05 ± 0,02%. In order to reveal whether these metabolic features were characteristic of the analyzed environment, the metagenomes from Ushuaia Bay were compared with 127 publicly available metagenomes, representative of diverse habitats including algae, seawater, freshwater, freshwater sediments, soil, human body microbiome as well as other marine and coastal sediments (S2 Table). Sequences assigned to biomarker genes for nitrification, DNRA and denitrification were found to be overrepresented in Ushuaia Bay with respect to the average abundance in these metagenomes (Fig 2A). For instance, sequences assigned to NosZ, catalyzing N_2_O reduction to N_2_, were 4.5 times more abundant in Ushuaia Bay sediments than the average of all metagenomes (Fig 2A), and almost twice with respect to the average for other marine sediments (Fig 2B). The same trend was observed for sequences assigned to Hao and NapA, which were approximately 6 times more abundant in Ushuaia Bay sediments than the general average, and twice to three times more abundant with respect to other marine sediments (S2 Fig). These results are compatible with an enhanced potential of the sediment microbial communities for the processing of free species of N, possibly fueled by the NH_4_^+^ available due to high allochthonous nutrient load and *in situ* degradation of organic matter [30].

In addition to well known biomarkers, we analyzed the abundance of sequences coding for accesory proteins NapB and NapC (two proteins acting as electron donors to NapA), NorC and NorE (accesory to nitrite oxide reductase NorB) following the same procedure than for the biomarker genes, using the corresponding KOs (K02568, K02569, K02305 and K02164, respectively). Notably, metagenomic sequences assigned to these accessory genes were detected in one order of magnitude lower abundance and with much higher variability than the biomarkers (average coefficient of variation for all genes and types of habitats: 107%, ranging from 13 to 700%), both in the dataset from Ushuaia Bay and in the 127 representative metagenomes used for comparison.

**Fig 3:**
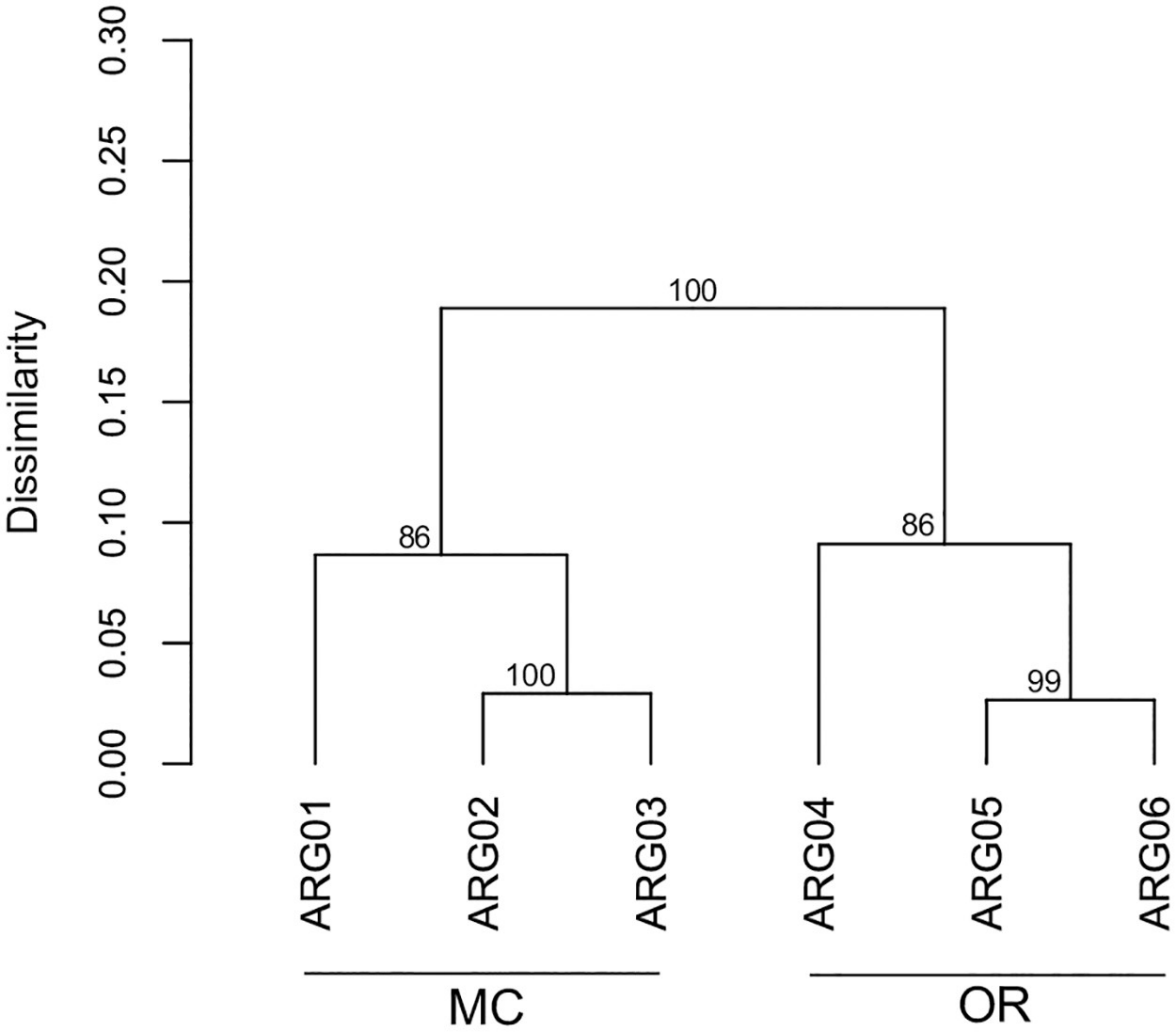
Cluster analysis of sediment metagenomes from Ushuaia Bay based on the abundance of sequences assigned to N-cycle biomarker genes. Bray-Curtis index was used as similarity measure. The grouping algorithm was average linkage. Bootstrap values were obtained from 1000 resamplings of the relative abundance matrix.

In order to evaluate whether there were differences in N-cycling footprints between the two analyzed sites in Ushuaia Bay, a multivariate analysis was performed based on the relative abundance of sequences assigned to the above mentioned biomarker genes. The average similarity between the N-cycling genetic patterns from MC and OR sites was high (85.9 ± 7.3%), yet clustering analysis resulted in two distinct groups according to sampling site (Fig 3). Based on the results of SIMPER analysis [41], sequences assigned to seven biomarkers (Hao, NirB, NarG, NapA NirK, NorB and NosZ) contributed 93% of the average dissimilarity between the structure of the dataset from the two sites, driving the observed grouping pattern (S3 Table). Moreover, the contribution of a single gene to the observed dissimilarity was relatively low (SIMPER values < 27%; S3 Table), suggesting that changes in abundance of a group of genes, rather than the predominance of a particular gene, were responsible for the detected grouping pattern. Among the mentioned biomarkers, sequences assigned to NarG, NirK, NosZ, and NirB (involved in denitrification and DNRA), were significantly more abundant in the metagenomes from samples of the MC site, while Hao (nitrification) was significantly more abundant in samples from the OR site (S1 Fig). The same trend was observed at the module level. Sequences assigned to the nitrification module (M00528) were more abundant in OR with respect to MC (0.07 ± 0.01% and 0.04 ± 0.01% of sequences assigned to modules, respectively), while denitrification (M00529) and DNRA (M00530) were higher in MC instead (0.62 ± 0.02 in MC vs. 0.45 ± 0.00 in OR for M00529; 0.49 ± 0.02 in MC vs. 0.39 ± 0.01 in OR for M00530).

In addition to biomarker genes, metagenomic sequences homologous to transcriptional regulators of N-cycle enzymes were searched in the dataset. The most abundant, although variable among samples, were sequences similar to the DNA-binding transcriptional regulator NorR (10^2^ to 10^3^ sequences per 10^7^ CDS). This regulator is known to participate in nitric oxide reduction, but also in nitric oxide detoxification and protection against reactive nitrogen intermediates [54]. Rrf2, also involved both in denitrification and detoxification of NO [55,56] was also represented (31 ± 16 sequences per 10^7^ CDS).

The typical *nos* operon regulator NosR [57] and FnrP, involved in *nos* regulation in atypical strains [58] were also well represented (50 ± 31 and 28 ± 21 sequences per 10^7^ CDS, respectively). NirI (regulator of *nir* operon, [59]) and NnrR, regulator of *nir* and *nor* operons in denitrification [60] were detected, although the latter in lower abundance (aproximately 24 ± 17 and 6 ± 3sequences per 10^7^ CDS, respectively).

### Diversity of putative enzymes related to denitrification in Ushuaia Bay sediments and their genomic context

The phylogenetic analysis of metagenomic sequences assigned to NirK and NosZ (10,334 and 28,181 sequences respectively) showed an extremely rich array of variants, widely distributed along the phylogeny known for each gene, and related to diverse bacterial lineages including Bacteroidetes, Chloroflexi, Firmicutes, Proteobacteria, Verrucomicrobia, as well as to phylotypes from the Archaea domain (S3 and S4 Figs). Interestingly, the majority of NosZ metagenomic sequences grouped within the clusters described as “atypical” [14] or Clade II [61], and included sequences similar to those found in genomes from *Sphaerobacter thermophilus* DSM 20745, *Rhodothermus marinus* DSM 4252, *Thiocapsa marina* 5811, *Magnetospirillum magneticum* AMB-1, *Dechloromonas aromatica* RC, *Gemmatimonas aurantiaca* T-27, *Psychroflexus torquis* ATCC 700755, *Maribacter* sp. HTCC2170, *Bizionia argentinensis* JUB59, *Marivirga tractuosa* DSM 4126, *Capnocytophaga gingivalis* ATCC 33624, and *Anaeromyxobacter* spp., among others (S4 Fig). In order to characterize this diversity at a finer scale, deduced amino acid sequences were grouped into OTUs based on their shared identity values. High OTU richness and diversity were found both for NirK and NosZ (Table 1). There were significant differences in NosZ diversity between sites: metagenomes from MC site showed higher diversity and lower dominance (Table 1). The same trend was observed for NirK, although no significant differences between sites could be detected in this case (Table 1).

**Table 1:** Alpha diversity metrics for NirK and NosZ in coastal sediments from Ushuaia Bay.

Target	Cutoff^&^	Site	Nseqs^†^	S_obs_^‡^	*Chao1*	H'^§^	Coverage	Dominance
NirK	0.17	MC	746	499 ± 29	1879 ± 290	8.53 ± 0.15	0.47 ± 0.04	0.005 ± 0.001
		OR		488 ± 20	1956 ± 197	8.40 ± 0.07	0.47 ± 0.03	0.006 ± 0.001
NosZ	0.20	MC	813	266 ± 21	612 ± 80	6.41 ± 0.20*	0.79 ± 0.03	0.04 ± 0.01*
		OR		250 ± 13	608 ± 92	5.62 ± 0.27*	0.79 ± 0.02	0.09 ± 0.03*

^&^Distance threshold at the amino acid level used to define OTUs.

^†^Number of sequences per sample used for analysis.

^‡^ Number of observed OTUs.

^§^Shannon-Wiener diversity index

*Significantly different between sites (Welch’s Two Sample t-test, α = 0.05).

**Fig 4:**
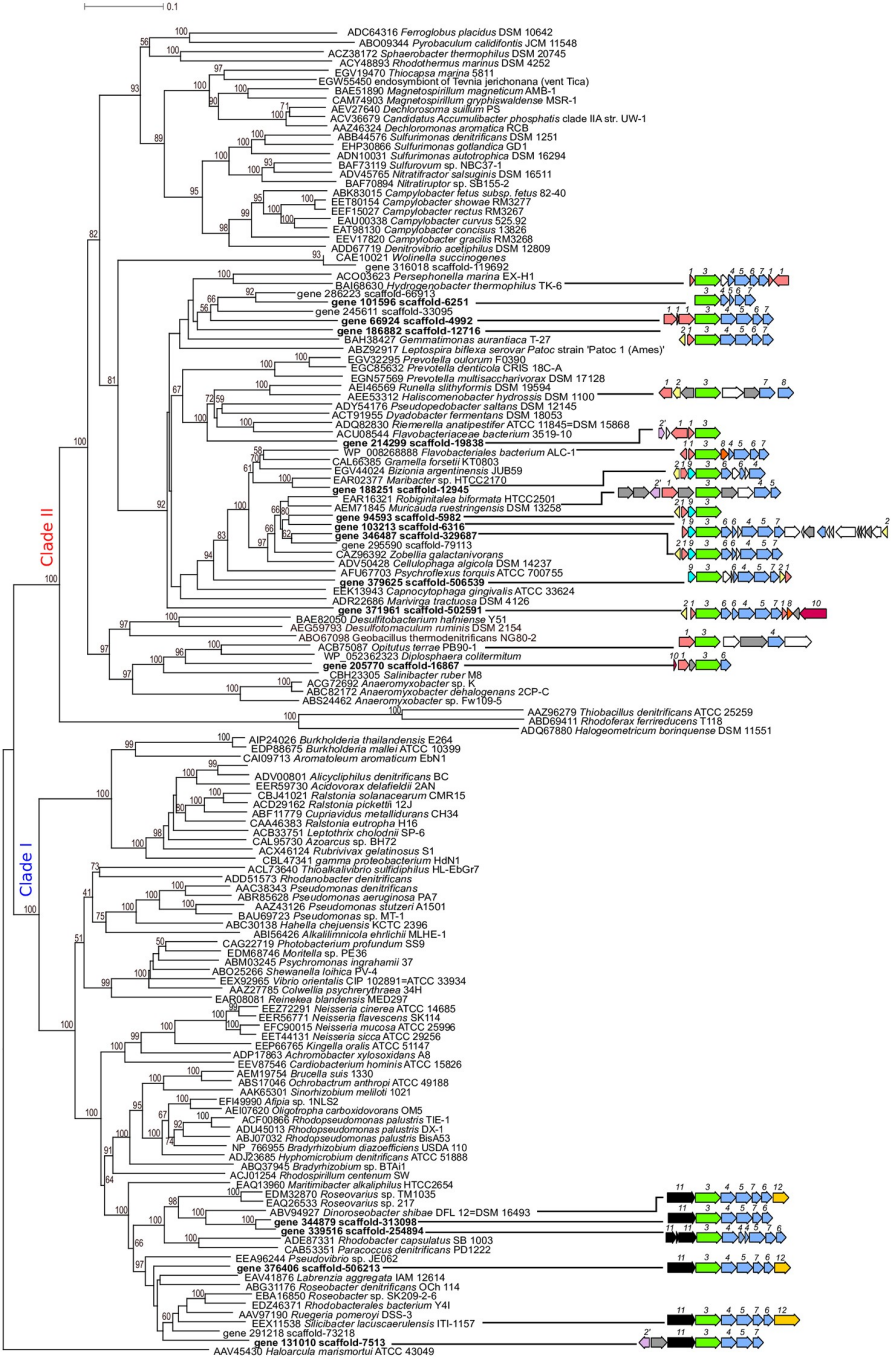
Phylogenetic tree of genomic/metagenomic NosZ sequences (left) and analysis of genomic context in scaffolds of a sediment metagenomic library from OR site (right), and related genomes. In the tree, genes from the metagenomic library are shown in bold, named with the gene number followed by the scaffold number. Bootstrap values are percent of 100 replications. In scaffold diagrams, numbers correspond to different genes, as assigned by RAST annotation system followed by manual curation. 1: c-type cytochrome; 2 and 2’: *rrf*2 and *nnr*R (Crp/Fnr family), respectively; 3: *nos*Z, 4: *nos*D; 5: *nos*F; 6: *nos*L; 7 *nos*Y; 8: transmembrane protein; 9: sensory subunit of low CO_2_ induced protein; 10: Iron-sulfur (4Fe-4S) protein; 11: *nos*R; 12: *nos*X. Genes encoding for other proteins or hypothetical proteins are colored grey or white, respectively.

Due to its environmental relevance in N_2_O emissions mitigation, NosZ constitutes a key biomarker of the denitrification potential. We were therefore interested in analyzing the genomic context and gene organization of *nosZ*, in order to gain insight into the evolutionary conservation of gene clusters associated to this capability. Unfortunately, due to the complexity of these microbial communities, assembly efficiency of metagenomes was low [37], and even the most abundant sequences assigned to NosZ were contained in short scaffolds or unassembled reads. Therefore, sequences from scaffolds carrying the *nos*Z gene were obtained from a fully-sequenced fosmid library constructed from intertidal sediments from OR site [33,49]. A total of 21 scaffolds were retrieved from the dataset, 15 of which harbored a complete *nos*Z gene and flanking sequences (*nos* clusters) (Table 2). In accordance with the results obtained from direct shotgun sequencing, the majority of *nos*Z sequences (73%) identified in the library grouped with Clade II variants, and were positioned mainly in the clade formed by sequences related to members of Bacteroidetes (Fig 4). Clade I variants (27%) were mainly related to sequences described in members of the Alphaproteobacteria. The taxonomic assignment of all the scaffolds (performed with PhylophythiaS, Table 2) were coincident at the broad phylogenetic level with the nearest neighbors of the NosZ sequences in the phylogenetic tree, suggesting that horizontal gene transfer, if any, was limited to members of phylogenetically-related groups.

**Table 2:** Characteristics of NosZ-carrying scaffolds in a metagenomic library constructed from coastal sediments from Ushuaia Bay. Only scaffolds with complete nosZ genes and at least one flanking coding sequence are shown.

Scaffold	bp^&^	CDS^†^	Taxonomic assignment	type of NosZ
4992	29632	29	Flavobacteriia, Flavobacteriales	Clade II
5982	37340	28	Flavobacteriia, Flavobacteriales	Clade II
6251	23988	22	Cytophagia, Cytophagales	Clade II
6316	40052	47	Flavobacteriia, Flavobacteriales	Clade II
7513	33543	39	Alphaproteobacteria, Rhodobacterales	Clade I
12716	21494	18	Bacteroidetes Order II. Incertae sedis	Clade II
12945	15515	17	Flavobacteriia, Flavobacteriales	Clade II
16867	4782	5	Verrucomicrobia, Spartobacteria, unclassified	Clade II
19838	25761	30	Gammaproteobacteria, Vibrionales	Clade II
254894	11144	12	Alphaproteobacteria, Rhodobacterales	Clade I
313098	8883	6	Alphaproteobacteria, Rhodobacterales	Clade I
329687	29683	33	Flavobacteriia, Flavobacteriales	Clade II
502591	34555	32	Actinobacteria, Actinomycetales	Clade II
506213	41600	31	Alphaproteobacteria, Rhodobacterales	Clade I
506539	43336	37	Bacteroidia,Bacteroidales	Clade II

^&^ Number of base pairs

^†^Number of predicted coding sequences

With regard to gene content, most of the *nos* clusters contained the four *nos*Z maturation genes: *nos*D, *nos*F, *nos*Y and *nos*L (Fig 4). Genes related to the detoxification of NO (*dnr*N, *nnr*S) were also found in some scaffolds (Fig 4). On the other hand, clear differences in gene content and organization between scaffolds carrying *nos*Z variants from different Clades could be observed. In particular, clusters carrying typical (Clade I) *nos* genes were very similar, presenting high shared synteny, while Clade II *nos*-carrying clusters were more variable. In addition, only Clade II *nos-*carrying clusters presented coding sequences assigned to c-type cytochromes and iron-sulfur proteins (4Fe-4S), which are potential electron transfer units [14]. Another difference was related to regulatory elements: Clade I *nos* clusters always presented the membrane-bound regulatory and electron transfer component *nos*R immediately upstream of *nos*Z, while Clade II *nos*-carrying scaffolds did not (Fig 4). On the contrary, the latter were enriched in transcriptional regulators from Rrf2 family (Fig 4). Notably, NnrR-like sequences were only found in two cases, one typical scaffold (7513, assigned to Alphaproteobacteria) and one atypical (19838 assigned to CFB superphylum) (Fig 4).

## Discussion

This study analyzes for the first time the microbial N-cycling potential in sediments from a subantarctic environment, Ushuaia Bay, using a metagenomic approach. Ushuaia Bay is located in Tierra del Fuego Island, south of Argentina, a region that has been increasingly impacted not only by recent climate change (e.g. glacier retreat, [62]) but also by local anthropogenic activities as a result of population growth [29,30]. This environmental context has likely influenced the native microbial communities of Ushuaia Bay sediments, which exhibit distinct patterns of adaptation, e.g to hydrocarbon pollution [31,33,38,63]. As previously reported, these communities were highly diverse [38], a feature which might limit the detection of biomarker genes related to key biogeochemical processes due to decreased coverage. This information, however, is relevant, as many key functional processes such as N_2_ fixation, ammonium oxidation, and methanogenesis, are often carried out by low-abundance microorganisms [64,65]. In this work, with a sequencing effort of one Illumina lane per sample, resulting in a final dataset of 2.5 to 16 Gbases per sample, it was possible to retrieve sequences assigned to most of the biomarkers related to N metabolism in these metagenomes. The only exception was NirS, which was unexpected since it has been found more often in marine sediments than its analogous NirK (e.g. by PCR-based methods, [66,67]). However, when non-PCR-dependent methods were used, sequences assigned to NirS were detected in very low abundance in temperate marine sediments, when compared with other denitrification-related biomarkers [28], suggesting that PCR biases could have affected previous estimations of *nir*S abundance. In contrast to biomarkers, the evaluated accessory genes were not detected consistently across metagenomes. This result could be due to the more limited sequence information available for these genes with respect to more studied biomarkers, which could have affected metagenomic functional annotation, especially in these environments characterized by poorly studied microbial communities with divergent genes [37, 38].

In this work, we found evidence that the genetic potential for the subprocesses catalyzing the biological transformations of ammonium and nitrate, including nitrification, DNRA and complete denitrification to N_2_ gas, is enhanced in Ushuaia Bay with respect to other environments, including other marine sediments, which typically tend to exhibit a strong nitrification-denitrification pattern [68]. Furthermore, the microbial communities from MC site (the Commercial pier), which is situated in a low water quality zone in the Bay affected by inputs of labile organic matter, and where net sediment NO_3_^-^ consumption has been previously observed [30] presented a higher abundance of NapA/NarG sequences (both coding for nitrate reductase), and higher abundance and diversity of NirK and NosZ variants compared to OR site. Both denitrification and DNRA are reductive processes starting from nitrate that provide energy and an electron sink for the respiration of organic matter (although some chemolitotrophs exist that are capable of DNRA, [12]. Accordingly, sequences assigned to NapA, which participates in both processes, were overrepresented. Genes coding for this enzyme have been shown to be ubiquitous, being more often present in genomes from environmental microorganisms than the enzymes of other steps of the N-cycle, which seem to be more restricted [17]. Moreover, in addition to nitrate reduction, this enzyme also has a role in redox homeostasis during aerobic respiration [69], and therefore the higher abundance of NapA could also be related to the adaptation of aerobic microorganisms to this suboxic environment. However, while DNRA retains N in a fixed form (ammonium), denitrification transforms nitrate to N_2_, removing N from the ecosystem, which in the environmental context of Ushuaia Bay can be considered as an ecosystem service [12,13,30]. In our study, although DNRA-specific biomarkers and their corresponding pathway modules were well represented in the metagenomes, they were always found to be less abundant than denitrification footprints, suggesting that, although both processes could be relevant, denitrification could be a dominant process in this ecosystem. It has been previously suggested that DNRA could be of less importance in cold sediments with respect to tropical latitudes [70,71]. In accordance to our results, in a recent metagenomic study of Baltic Sea and North Sea, DNRA was shown as a well represented process, although significantly lower than denitrification [28]. The pattern was attributed to conditions of high N availability (low C:N ratio), which are thought to favor denitrification over DNRA [12,72]. It has to be noted, however, that both the mentioned study of temperate marine sediments [28] and this work are based on estimated gene abundances in metagenomic datasets. Further studies involving transcription and metabolic fluxes would be needed to confirm these results.

Denitrification-related biomarker genes identified in the sediment metagenomes were affiliated to a wide range of microbial phyla. In the case of NosZ, Clade II variants were the main contributors to this phylogenetic diversity. These sequences were more abundant in both analyzed datasets (the direct shotgun sequencing dataset and a metagenomic fosmid library) with respect to typical (Clade I) variants. In addition, they presented a more variable genomic context. This is in accordance with their wider phylogenetic range: while Clade I *nos*Z are known to be carried by Alpha-, Beta-, and Gammaproteobacteria, the evolutionary history of Clade II clusters seems to originate deeper, covering various bacterial phyla, including the bacterial lineages Aquificae, Chloroflexi, Bacteroidetes, Verrucomicrobia, and Crenarchaeota (Archaea), among others [14,73]. There is increasing evidence that populations carrying Clade II *nos* variants are abundant in a variety of environments and could represent potentially underestimated players in the mitigation of N_2_O emissions [14,74–76]. In particular, N_2_O reducers with Clade II NosZ have been found in a wider range of habitats with more extreme environmental conditions [14]. Genome analysis and cultured-based studies suggest that the ecophysiology of N_2_O reduction can differ between the populations carrying the two clades [73]. Many of Clade II *nosZ*-carrying microorganisms are not capable of performing the previous denitrification steps, as they lack the corresponding genes (*nirK* or *nirS*), indicating that they are independently utilizing N_2_O for respiration (“non-denitrifying N_2_O reducers”). Moreover, Clade II NosZ enzymes have higher affinity for N_2_O than their Clade I counterparts, and their host microbes (*Dechloromonas aromatica* strain RCB and *Anaeromyxobacter dehalogenans* strain 2CP-C) produce more biomass per mole of N_2_O consumed [77], indicating the key environmental role that enzymes with low Ks from these populations could have in regulating N_2_O emmisions. Others, however, exhibit different strategies of N_2_O use. *Gemmatimonas aurantiaca* T-27, an obligate aerobe bacterium, showed N_2_O consumption both in the presence and absence of oxygen, but needed oxygen for *nosZ* transcription and the use of N_2_O was transient and not coupled to growth, indicating a role of N_2_O as a surrogate electron acceptor for cell maintenance during oxygen starvation periods in suboxic enviroments [78]. Aerobic NosZ-carrying microorganisms are an important portion of the community in various environments, and their role in N_2_O removal could be key in environments which alternate between oxic and anoxic conditions, such as subsurface soils or surficial sediments. The results from this work showed the presence in the metagenomes of genes very similar to all of the mentioned populations and many other yet-to-be characterized variants, indicating that the depuration of toxic forms of N in this subantarctic environment is likely to be carried out by various members of the microbial community with very different lifestyles, increasing the likelihood for resilience against disturbance [79].

Due to the fact that the energetic yield of oxygen respiration is higher than the one for nitrate, denitrification is in many cases regulated to be dominant only at limiting oxygen concentrations [80]. Transcriptional as well as post-transcriptional controls are known to occur, such as the inhibition of nitrate transport by oxygen, and a number of regulatory mechanisms targeting genes for the different denitrification steps [80,81]. Over the last decades, our knowledge of the regulation of the denitrification process has increased, mainly by studies in model organisms such as *Pseudomonas stutzeri* or *Paracoccus denitrificans* [80]. However, there is still limited information regarding these regulations in environmentally relevant N_2_O-reducing microorganisms [73,82], especially in groups other than Proteobacteria [83]. Based on the current knowledge on model organisms, transcriptional control is mainly carried out by three members of the FNR/CRP superfamily of transcriptional regulators, which respond to environmental (e.g. O_2_, NO_3_^-^, NO) as well as metabolic signals [80]. All of these regulators were present in the shotgun dataset, and the high abundance of regulators that participate also in detoxification such as *nor*R, highlights the need of the microbial community to cope with toxic forms of nitrogen. When analyzing the scaffolds of the metagenomic library, there were clear differences in the type of regulatory elements found in variants from different clades. Sequences identified as *nos*R were found to be consistently present in scaffolds containing Clade I *nosZ* genes, which were assigned mainly to Alphaproteobacteria. NosR is a membrane-bound regulatory component which is not only involved in copper-dependent transcription of the *nos* operon, but also necessary for sustaining NosZ activity, likely via electron donation [57,84,85]. This protein has been observed in model organisms, and also in genomic studies (associated with in Clade I *nosZ* variants) [14;73]. On the other hand, in Clade I *nos*-carrying clusters, other sequences identified as putative regulatory elements were present, namely related to Rrf2, a family of transcriptional regulators found in a number of genomes from bacteria belonging to Proteobacteria and Firmicutes [83]. These molecules control a variety of steps of denitrification as well as NO detoxification in these microorganisms, and respond to NO_2_ and NO. However, this information is limited to the previously mentioned phylogenetic groups, and it is known that regulation of this process varies widely with phylogeny, so it is difficult to predict the role of these molecules in Clade II variants for which no cultured model is available [83]. As discussed by Hallin et al. [61], more exhaustive biochemical and physiological studies are required to determine how differences in gene arrangement and regulation of *nos* clusters result in physiological differences, as this could potentially drive niche differentiation between host populations.

This in-depth metagenomic work aimed to contribute to the comprehension of N-cycling potential in microbial communities of coastal sediments from subantarctic environments. This work presents a full description of genomic signatures associated with major N-metabolic subprocesses, despite the limitations associated with metagenomic analyses of high complexity environments, such as problems in coverage and assembly efficiency. Our results suggest that denitrification could be the major driver of N-cycling, in accordance to the environmental perturbation of this coastal area, impacted by organic matter, high nutrient load and hydrocarbon pollution, within a low-energy environment. The denitrification pathway was found to be completely represented by sequences assigned to the enzymes needed for N_2_ production, accompanied by a high diversity of nitrous oxide reductase-associated sequences. Overall, these results suggest that these communities could contribute to the alleviation of harmful nitrogen species like N_2_O released from euthrophicated coastal environments into the atmosphere.

## Supporting information

S1 Fig Relative abundance of sequences assigned to biomarker genes for N-cycling in sediments from Ushuaia Bay, discriminated by sampling site. Numbers correspond to percent amino acid sequences, with respect to total sequences assigned to KOs. Differences in abundances between sites were evaluated by Welch two-sample test (α = 0.05). *p-value <0.05; **p-value <0.01, ***p-value <0.001.
(PDF)

S2 Fig Relative abundance of the sequences assigned to Hao and NapA in various metagenomes, discriminated by type of environment. Metagenomic sequences were annotated in the IMG/M pipeline. Abundances of specific KEGG orthology (KO) identifiers were retrieved as “estimated gene copies” (assembled and unassembled fractions), normalized to the total sequences assigned to KOs. For details of the 127 metagenomes used for comparisons, see S2 Table.
(PDF)

S3 Fig Evolutionary placement analysis of putative NirK sequences identified in the metagenomes of Ushuaia Bay sediments. The tree was constructed by Maximum Likelihood in RAxML v.8.2.3. Reference sequences are indicated by GenBank accession numbers followed by title. Metagenomic sequences are indicated with blue and red colored branches, for sediment samples from OR and MC sites, respectively. The width of the colored branches is proportional to the abundance of metagenomic sequences assigned to that branch. Only bootstrap values > 50% are shown.
(PDF)

S4 Fig Evolutionary placement analysis of putative NosZ sequences identified in the metagenomes of Ushuaia Bay sediments. The tree was constructed by Maximum Likelihood in RAxML v.8.2.3. Reference sequences are indicated by GenBank accession numbers followed by title. Metagenomic sequences are indicated with blue and red colored branches, for OR and MC, respectively. The width of the colored branches is proportional to the abundance of metagenomic sequences assigned to that branch. Only bootstrap values > 50% are shown.
(PDF)

S1 Table Physicochemical characteristics of Ushuaia Bay sediment samples. Results are expressed as mean and standard deviation of 10 measurements, with the exception of TOM and NH_4_^+^, which were measured in triplicate.
(PDF)

S2 Table Abundance of sequences assigned to genes encoding N-cycling enzymes selected for this study. “Estimated gene copies, assembled and unassembled metagenomes” were obtained from sequences assigned to KO identifiers at the IMG database (www.img.jgi-doe.gov).
(PDF)

S3 Table Similarity percentage (SIMPER) analysis of sediment metagenomes based on abundance of amino acid sequences assigned to N-cycling biomarker genes, grouped by site. Bold types indicate biomarkers whose cumulative contribution to the relative dissimilarity was ≥ 90%.
(PDF)
