# Supplementary figures and images for: Predominance and high diversity of genes associated to denitrification in metagenomes of subantarctic coastal sediments exposed to urban pollution
Source: PLoS One. 2018 Nov 29;13(11):e0207606. doi: 10.1371/journal.pone.0207606 (PMC6264515; doi:10.1371/journal.pone.0207606)

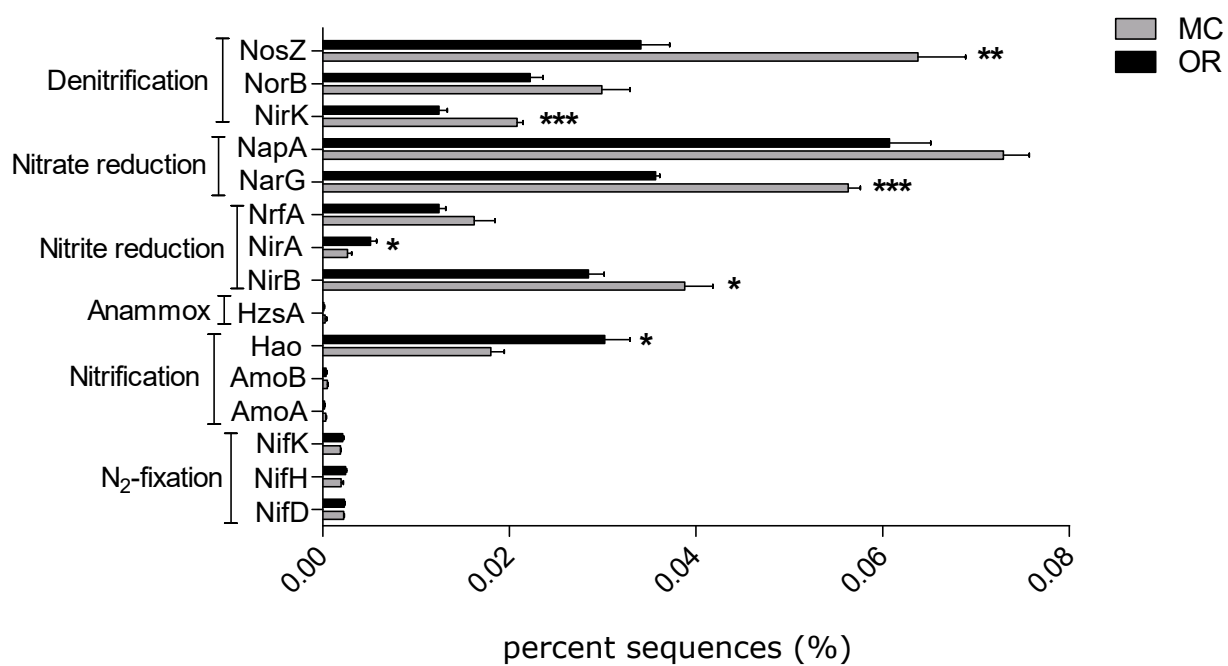

S1 Fig

Supplement: S1 Fig — Numbers correspond to percent amino acid sequences, with respect to total sequences assigned to KOs. Differences in abundances between sites were evaluated by Welch two-sample test (α = 0.05). *p-value <0.05; **p-value <0.01, ***p-value <0.001. (PDF) [file pone.0207606.s001.pdf]

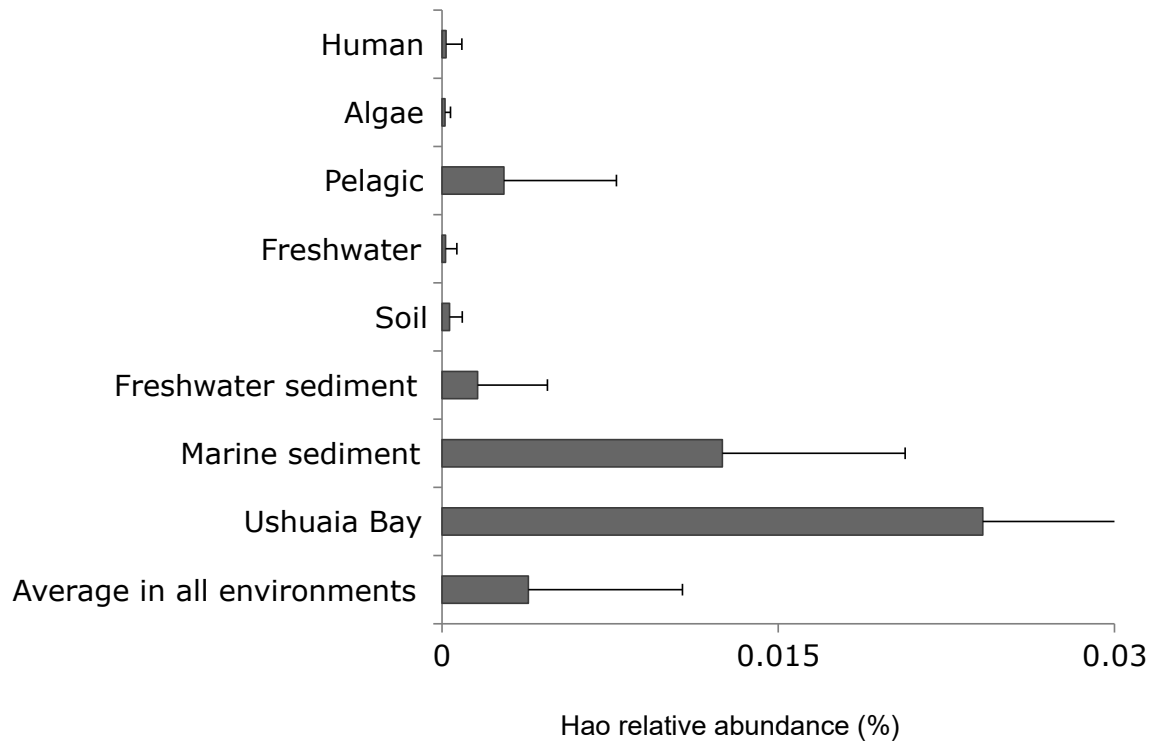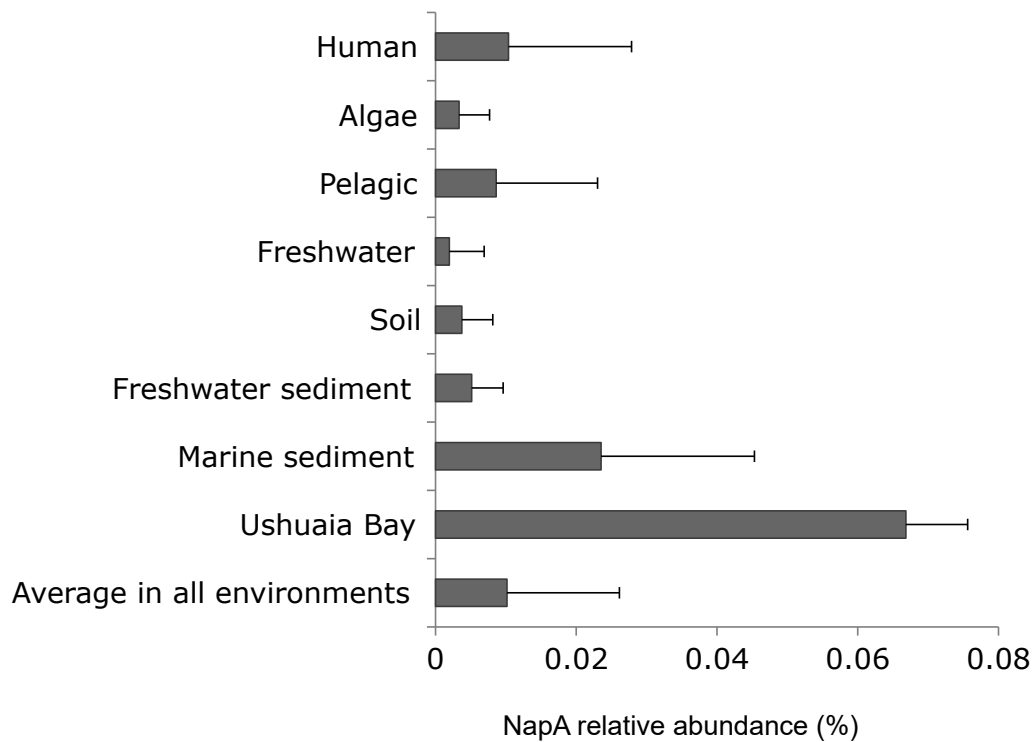

S2 Fig

Supplement: S2 Fig — Metagenomic sequences were annotated in the IMG/M pipeline. Abundances of specific KEGG orthology (KO) identifiers were retrieved as “estimated gene copies” (assembled and unassembled fractions), normalized to the total sequences assigned to KOs. For details of the 127 metagenomes used for comparisons, see S2 Table. (PDF) [file pone.0207606.s002.pdf]

# S4 Fig

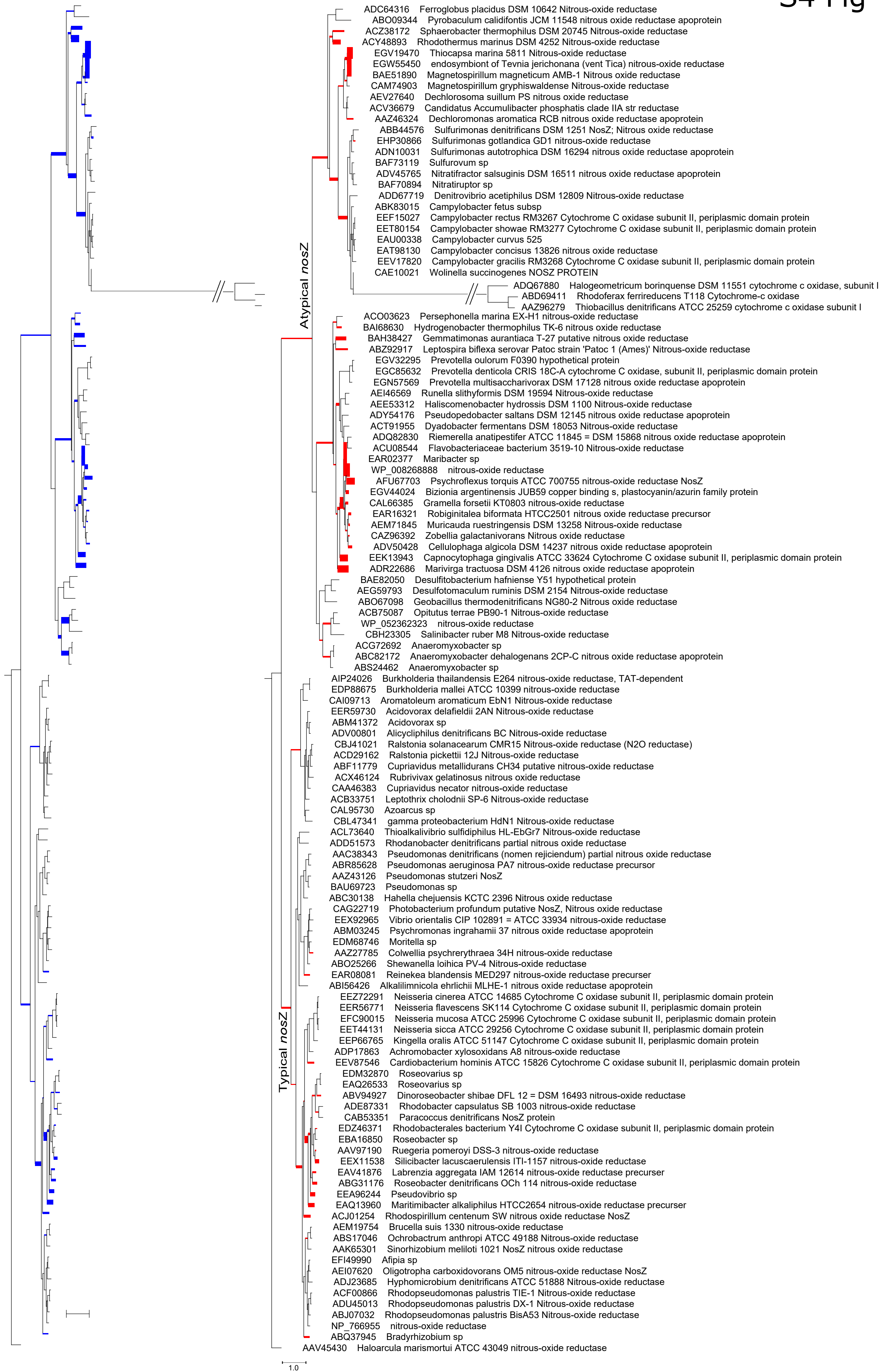

OR  
MC

Supplement: S4 Fig — The tree was constructed by Maximum Likelihood in RAxML v.8.2.3. Reference sequences are indicated by GenBank accession numbers followed by title. Metagenomic sequences are indicated with blue and red colored branches, for OR and MC, respectively. The width of the colored branches is proportional to the abundance of metagenomic sequences assigned to that branch. Only bootstrap values > 50% are shown. (PDF) [file pone.0207606.s004.pdf]
