# Supplementary material for: Predominance and high diversity of genes associated to denitrification in metagenomes of subantarctic coastal sediments exposed to urban pollution
Source: PLoS One. 2018 Nov 29;13(11):e0207606. doi: 10.1371/journal.pone.0207606 (PMC6264515; doi:10.1371/journal.pone.0207606)
Supplement: S1 Table — Results are expressed as mean and standard deviation of 10 measurements, with the exception of TOM and NH4+, which were measured in triplicate. (PDF) [file pone.0207606.s005.pdf]

Table S1. Physicochemical characteristics of Ushuaia Bay sediment samples. Results are expressed as mean and standard deviation of 10 measurements, with the exception of TOC and NH_4^+, which were measured in triplicate.

Site	Lat/Lon	Sample	Depth (m)	Temp (°C)	Sal (%)	pH	TOM (%)	Total NH_4^+ (ng/g ws)
MC	54° 48.7' S 68° 17.7' W	ARG01	11.3	8.52 ± 0.04	29.42 ± 0.04	8.00 ± 0.00	0.13 ± 0.01	1462 ± 92
		ARG02	11.3	8.66 ± 0.05	29.34 ± 0.05	8.01 ± 0.01	0.15 ± 0.01	1192 ± 33
		ARG03	11.3	8.50 ± 0.00	29.42 ± 0.00	7.99 ± 0.00	0.20 ± 0.03	1997 ± 24
OR	54° 48.3' S 68° 17.3' W	ARG04	12.3	8.74 ± 0.05	29.24 ± 0.05	7.68 ± 0.05	nd	nd
		ARG05	12.3	8.60 ± 0.00	29.30 ± 0.00	7.79 ± 0.01	0.30 ± .0.05	2129 ± 88
		ARG06	12.3	8.60 ± 0.00	29.30 ± 0.00	7.84 ± 0.01	0.74 ± 0.02	3134 ± 147

Abbreviations: Sal: salinity, TOM: total organic matter content, nd: non -determined, ws: wet sediment.
