# Supplementary material for: Predominance and high diversity of genes associated to denitrification in metagenomes of subantarctic coastal sediments exposed to urban pollution
Source: PLoS One. 2018 Nov 29;13(11):e0207606. doi: 10.1371/journal.pone.0207606 (PMC6264515; doi:10.1371/journal.pone.0207606)
Supplement: S2 Table — “Estimated gene copies, assembled and unassembled metagenomes” were obtained from sequences assigned to KO identifiers at the IMG database (www.img.jgi-doe.gov). (PDF) [file pone.0207606.s006.pdf]

Table S2. Biomarker genes encoding N-cycling enzymes selected for this study and their abundances in different habitats. Estimated gene copies (assembled and unassembled fractions of the metagenomes) were obtained from sequences assigned to KO identifiers (ID) at IMG/M.

		Estimated gene copies (mean ± SD)								
	KO ID	This study	Marine sediment	Freshwater sediment	Soil	Freshwater	Seawater	Algae	Human body	General average
N-fixation	NifH (K02588)	0.0022 ± 0.0004	0.0101 ± 0.0292	0.0038 ± 0.0055	0.0051 ± 0.0183	0.0053 ± 0.0119	0.0011 ± 0.0025	0.0001 ± 0.0001	0.0030 ± 0.0064	0.0050 ± 0.0174
	NifD (K02586)	0.0023 ± 0.0001	0.0101 ± 0.2920	0.0038 ± 0.0055	0.0051 ± 0.0183	0.0053 ± 0.0119	0.0011 ± 0.0025	0.0004 ± 0.0012	0.0030 ± 0.0064	0.0050 ± 0.1740
	NifK (K02591)	0.0020 ± 0.0002	0.0074 ± 0.0223	0.0059 ± 0.0112	0.0066 ± 0.0263	0.0055 ± 0.0134	0.0012 ± 0.0027	0.0008 ± 0.0024	0.0003 ± 0.0012	0.0047 ± 0.0169
Nitrification	AmoA (K10944)	0.0003 ± 0.0001	0.0003 ± 0.0004	0.0059 ± 0.0208	0.0087 ± 0.0259	0.0275 ± 0.1012	0.0017 ± 0.0036	0.00003 ± 0.0001	-	0.0056 ± 0.0360
	AmoB (K10945)	0.0005 ± 0.0001	0.0004 ± 0.0005	0.0007 ± 0.0009	0.0039 ± 0.0100	0.0293 ± 0.1081	0.0024 ± 0.0046	0.0001 ± 0.0002	-	0.0045 ± 0.0361
	Hao (K10535)	0.0241 ± 0.0075	0.0125 ± 0.0082	0.0016 ± 0.0031	0.0003 ± 0.0006	0.0002 ± 0.0005	0.0028 ± 0.0050	0.0001 ± 0.0002	0.0002 ± 0.0007	0.0039 ± 0.0069
Anammox	HzsA	0.0002 ± 0.0002*	nd	nd	nd	nd	nd	nd	nd	nd
Assimilatory nitrite reduction	NirA (K00366)	0.0039 ± 0.0016	0.0079 ± 0.0124	0.0165 ± 0.0225	0.0282 ± 0.0414	0.0069 ± 0.0149	0.0045 ± 0.0070	0.0060 ± 0.0113	0.0043 ± 0.0058	0.0115 ± 0.0227
Nitrite reduction to ammonia	NirB (K00362)	0.0336 ± 0.0068	0.0244 ± 0.0155	0.0212 ± 0.0172	0.0309 ± 0.0367	0.0107 ± 0.0153	0.0125 ± 0.0172	0.0387 ± 0.0346	0.0087 ± 0.0183	0.0211 ± 0.0243

Dissimilatory nitrate reduction to ammonia	NrfA (K03385)	0.0144 ± 0.0033	0.0102 ± 0.0069	0.0079 ± 0.0093	0.0034 ± 0.0044	0.0032 ± 0.0074	0.0014 ± 0.0024	0.0009 ± 0.0013	0.0186 ± 0.0222	0.0007 ± 0.0108
Dissimilatory nitrate reduction to nitrite	NapA (K02567)	0.0668 ± 0.0088	0.0235 ± 0.0218	0.0051 ± 0.0045	0.0037 ± 0.0044	0.0020 ± 0.0049	0.0086 ± 0.0144	0.0034 ± 0.0043	0.0104 ± 0.0175	0.0102 ± 0.0160
	NarG (K00370)	0.0460 ± 0.0114	0.0346 ± 0.0252	0.0292 ± 0.0176	0.0535 ± 0.1519	0.0040 ± 0.0041	0.0153 ± 0.0219	0.0018 ± 0.0021	0.0140 ± 0.0226	0.0265 ± 0.0681
Denitrification	NirS (K15864)	-	0.0010 ± 0.0030	0.0036 ± 0.0062	0.0009 ± 0.0026	0.0006 ± 0.0013	0.0050 ± 0.0103	0.0008 ± 0.0017	-	0.0017 ± 0.0050
	NirK (K00368)	0.0167 ± 0.0047	0.0124 ± 0.0111	0.0121 ± 0.0068	0.0268 ± 0.0385	0.0024 ± 0.0020	0.0075 ± 0.0123	0.0051 ± 0.0090	0.0055 ± 0.0093	0.0119 ± 0.0198
	NorB (K04561)	0.0261 ± 0.0056	0.0204 ± 0.0147	0.0158 ± 0.0129	0.0200 ± 0.0240	0.0027 ± 0.0063	0.0063 ± 0.0106	0.0038 ± 0.0064	0.0122 ± 0.0238	0.0137 ± 0.0175
	NosZ (K00376)	0.0489 ± 0.0175	0.0265 ± 0.0205	0.0073 ± 0.0047	0.0054 ± 0.0064	0.0009 ± 0.0008	0.0110 ± 0.0184	0.0014 ± 0.0024	0.0043 ± 0.0095	0.0108 ± 0.0161

Nitrogen fixation: NifH Nitrogenase iron protein, NifD Nitrogenase molybdenum-iron protein alpha chain and NifK Nitrogenase molybdenum-iron protein beta chain. Nitrification: AmoA Ammonia monooxygenase subunit A, AmoB Ammonia monooxygenase subunit B and Hao Hydroxylamine dehydrogenase. Anammox: HzsA Hydrazine synthase. Assimilatory nitrite reduction: NirA Ferredoxin-nitrite reductase. Nitrite reduction to ammonia: NirB NAD(P)H nitrite reductase. Dissimilatory nitrate reduction to ammonia: NrfA cytochrome c nitrite reductase. Dissimilatory nitrate reduction: NapA Periplasmic nitrate reductase and NarG Bound-membrane nitrate reductase alpha subunit. Denitrification: NirS Cytochrome cd1-type nitrite reductase, NirK Cu-containing nitrite reductase, NorB Nitric oxide reductase subunit B and NosZ Nitrous-oxide reductase.

*Amino acid sequences were retrieved by searching against inhouse-built HMM (for details see Materials and Methods).

Accession numbers of the metagenomes obtained from IMG/M are as follows. Marine sediment: 3300000884, 3300001687, 3300005809, 3300003145, 3300003144, 3300003141, 3300002242, 3300002231, 3300001854, 3300000511, 3300001136, 3300000515, 3300000374, 3300000426, 2199352009, 3300000127, 3300000133, 3300000120, 3300000130, 3300000128, 3300000243, 3300000136, 3300000129, 3300000132, 3300000123, 3300000119, 3300000135, 3300000792, 3300000134, 3300000124, 3300000241, and 3300000126. Freshwater sediment: 3300003087, 3300012232, 3300009610, 3300009609, 3300009075, 3300009146, 3300003684,

3300002161, 3300002184, 3300003754, 3300002447, 3300003152, 3300006052, 3300006162, 3300006050, and 3300012931. Soil: 3300002840, 3300005530, 3300005341, 3300001150, 3300000793, 3300000907, 3300001632, 3300003505, 3300010896, 3300010364, 2044078000, 3300005271, 3300001422, 3300011015, 3300003729, 3300003241, 2070309009, 3300000335, 3300005294, 3300000661, 3300012971, 3300003725, 3300012514, and 3300005610. Freshwater: 3300011180, 3300004124, 3300003488, 3300005582, 3300003686, 3300004807, 3300007522, 3300009684, 3300007516, 3300002348, 3300009068, 3300012774, 3300000882, and 2049941002. Seawater: 2008193000, 2006543006, 3300001940, 3300001938, 3300001939, 3300001941, 3300001944, 3300001948, 3300001974, 3300009706, 3300001957, 3300001969, 2014613002, 3300009433, 3300001750, 3300001685, 3300002180, and 3300002225. Algae: 3300002062, 3300009073, 3300009446, 3300009192, 3300009415, 3300002037, 3300002057, and 3300002055. Human body: 2018322000, 7000000717, 7000000133, 3300003080, 3300001602, 3300002430, 3300003146, 3300003118, 7000000555, 7000000439, 7000000150, 7000000446, 7000000462, 7000000138, and 3300003109.
