# Supplementary material for: Predominance and high diversity of genes associated to denitrification in metagenomes of subantarctic coastal sediments exposed to urban pollution
Source: PLoS One. 2018 Nov 29;13(11):e0207606. doi: 10.1371/journal.pone.0207606 (PMC6264515; doi:10.1371/journal.pone.0207606)
Supplement: S3 Table — Bold types indicate biomarkers whose cumulative contribution to the relative dissimilarity was ≥ 90%. (PDF) [file pone.0207606.s007.pdf]

Table S3. Similarity percentage (SIMPER) analysis of sediment metagenomes based on abundance of amino acid sequences assigned to N-cycling biomarker genes, grouped by site. Bold types indicate biomarkers whose cumulative contribution to the relative dissimilarity was ≥ 90%.

Biomarker	Average abundance		Ordered cumulative contribution (%)
	MC	OR	
**NosZ**	**0.0638**	0.0341	27.2
**NarG**	**0.0563**	0.0357	46.2
**NapA**	**0.0729**	0.0607	57.8
**Hao**	0.0180	**0.0302**	68.9
**NirB**	**0.0388**	0.0285	78.3
**NirK**	**0.0208**	0.0125	86.1
**NorB**	**0.0299**	0.0222	93.1
NrfA	0.0162	0.0125	96.4
NirA	0.0027	0.0051	98.7
NifH	0.0020	0.0025	99.2
NifK	0.0019	0.0022	99.4
HszA	0.0003	0.0002	99.6
AmoB	0.0005	0.0004	99.7
NifD	0.0022	0.0023	99.9
AmoA	0.0004	0.0002	100
