# Supplementary material for: Predominance and high diversity of genes associated to denitrification in metagenomes of subantarctic coastal sediments exposed to urban pollution
Source: PLoS One. 2018 Nov 29;13(11):e0207606. doi: 10.1371/journal.pone.0207606 (PMC6264515; doi:10.1371/journal.pone.0207606)

S3 Fig

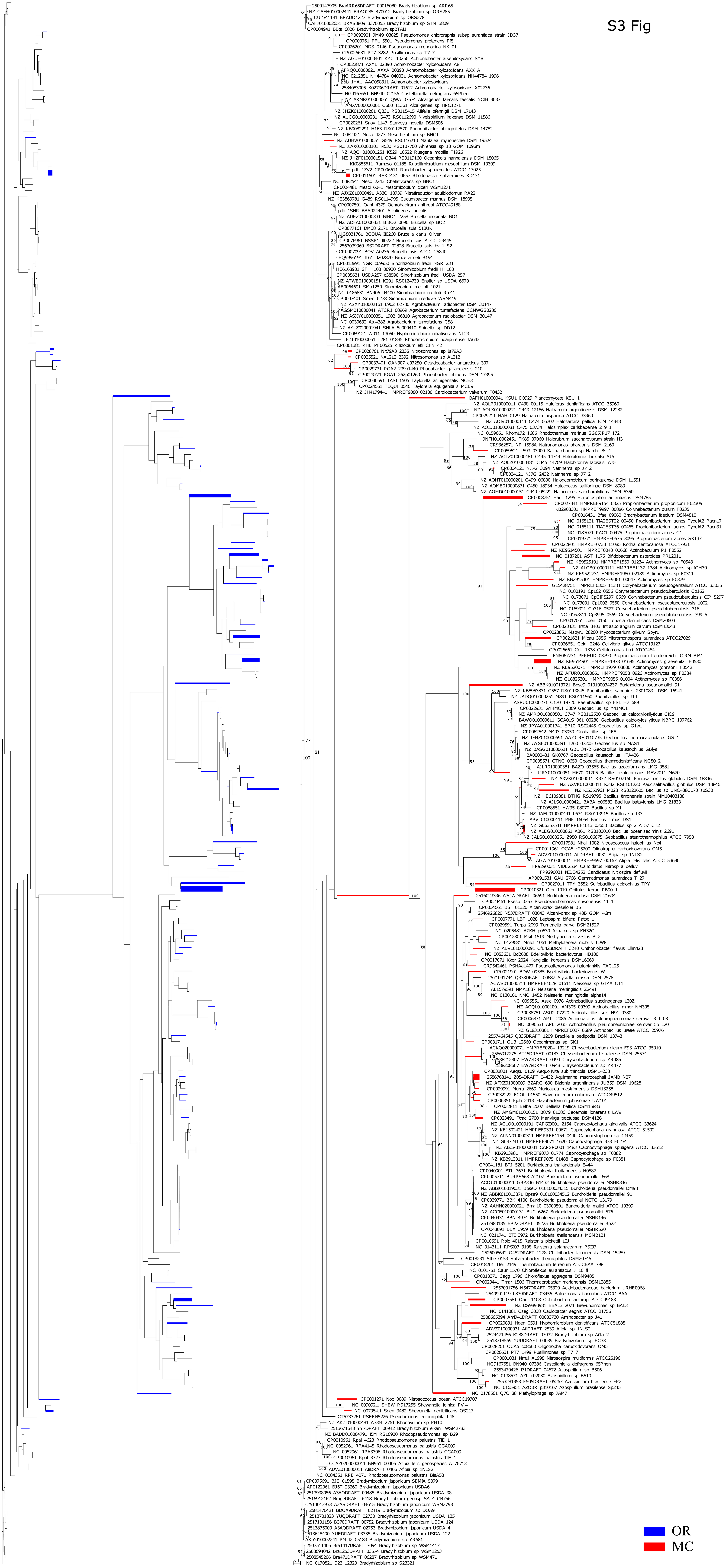

Supplement: S3 Fig — The tree was constructed by Maximum Likelihood in RAxML v.8.2.3. Reference sequences are indicated by GenBank accession numbers followed by title. Metagenomic sequences are indicated with blue and red colored branches, for sediment samples from OR and MC sites, respectively. The width of the colored branches is proportional to the abundance of metagenomic sequences assigned to that branch. Only bootstrap values > 50% are shown. (PDF) [file pone.0207606.s003.pdf]
